# Neutralizing GM-CSF autoantibodies in pulmonary alveolar proteinosis, cryptococcal meningitis and severe nocardiosis

**DOI:** 10.1186/s12931-022-02103-9

**Published:** 2022-10-11

**Authors:** Hélène Salvator, Aristine Cheng, Lindsey B. Rosen, Peter R. Williamson, John E. Bennett, Anuj. Kashyap, Li Ding, Kyung J. Kwon-Chung, Ho Namkoong, Christa S. Zerbe, Steven M. Holland

**Affiliations:** 1 Laboratory of Clinical Immunology and Microbiology, Division of Intramural Research, National Institute of Allergy and Infectious Diseases, National Institutes of Health, Bethesda, MD USA; 2 Present Address: Department of Respiratory Medicine, Hôpital Foch, Suresnes, France-UMR 0892 VIM Suresnes, INRAE Paris Saclay University, Jouy-en-Josas, France; 3 Present Address: Division of Infectious Diseases, Department of Medicine, National Taiwan University Hospital and National Taiwan University College of Medicine, Taipei, Taiwan; 4 Present Address: Department of Analytical Sciences, BioPharmaceuticals Development, BioPharmaceuticals R&D, AstraZeneca, Gaithersburg, MD USA; 5 Present Address: Department of Infectious Diseases, Keio University School of Medicine, Tokyo, Japan

**Keywords:** GM-CSF, Autoantibody, Pulmonary alveolar proteinosis, *Cryptococcus*, *Nocardia*

## Abstract

**Background:**

Anti GM-CSF autoantibodies (aAb) have been related to acquired pulmonary alveolar proteinosis (PAP) and described in cases of severe infections such as cryptococcosis and nocardiosis in previously healthy subjects. Whether there are different anti-GM-CSF autoantibodies corresponding to these phenotypes is unclear. Therefore, we examined anti-GM-CSF autoantibodies to determine whether amount or neutralizing activity could distinguish between groups.

**Methods:**

Plasma samples gathered in the National Institute of Health from patients with anti GM-CSF aAb and either PAP (n = 15), cryptococcal meningitis (n = 15), severe nocardiosis (n = 5) or overlapping phenotypes (n = 6) were compared. The relative amount of aAb was assessed using a particle-based approach, reported as a mouse monoclonal anti-human GM-CSF as standard curve and expressed in an arbitrary Mouse Monoclonal Antibody Unit (MMAU). The neutralizing activity of the plasma was assessed by inhibition of GM-CSF-induced intracellular phospho-STAT5 (pSTAT5) in monocytes.

**Results:**

Anti-GM-CSF aAb relative amounts were higher in PAP patients compared to those with cryptococcosis (mean 495 ± 464 MMAU *vs* 197 ± 159 MMAU, *p* = 0.02); there was no difference with patients with nocardiosis (430 ± 493 MMAU) nor between the two types of infections.

The dilution of plasma resulting in 50% inhibition of GM-CSF-induced pSTAT5 (approximate IC_50_) did not vary appreciably across groups of patients (1.6 ± 3.1%, 3.9 ± 6% and 1.8 ± 2.2% in PAP patients, cryptococcosis and nocardiosis patients, respectively). Nor was the concentration of GM-CSF necessary to induce 50% of maximal GM-CSF-induced pSTAT5 in the presence of 10 MMAU of anti-GM-CSF aAb (EC_50_). When studying longitudinal samples from patients with PAP or disseminated nocardiosis, the neutralizing effect of anti-GM-CSF aAb was relatively constant over time despite targeted treatments and variations in aAb levels.

**Conclusions:**

Despite different clinical manifestations, anti-GM-CSF antibodies were similar across PAP, cryptococcosis and nocardiosis. Underlying host genetics and functional analyses may help further differentiate the biology of these conditions.

## Background

Anti-cytokine autoantibodies are emerging entities that may explain some severe infections in previously healthy adults [1]. The most recent example is anti-type I interferon autoantibodies in patients with life-threatening COVID-19 [2]. Neutralizing anti-IFNy autoantibodies have also been shown to be responsible for disseminated mycobacterial diseases [3, 4], anti-interleukin (IL)-6 autoantibodies for staphylococcal skin infections, and anti-IL-17A/F autoantibodies for mucocutaneous candidiasis, [5] even though this latter association has recently been challenged [6]. These cases can be considered autoimmune phenocopies of previously recognized monogenic inborn errors, whose phenotypes are derived from Mendelian disruptions in the same pathways with the same pathogens [7].

However, the case of anti-granulocyte–macrophage-colony-stimulating-factor (anti-GM-CSF) autoantibodies appears to be more complex. Anti-GM-CSF antibodies were first described in 1999 and are now recognized as the main cause of acquired pulmonary alveolar proteinosis [8]. In pulmonary macrophages, GM-CSF receptor-ligand interaction induces signal transduction, notably via signal transducer and activator of transcription (STAT)-5 phosphorylation and induction of the transcription factor PU.1, an essential factor for surfactant catabolism and local pulmonary immunity [9]. Disruption of GM-CSF signaling leads to alveolar macrophage dysfunction, intra-alveolar accumulation of lipoproteinaceous material and the insidious onset of interstitial lung disease [10].

Neutralizing anti-GM-CSF autoantibodies have also been described as risk factors for disseminated infections such as cryptococcal meningitis and disseminated nocardiosis in previously healthy subjects [11, 12]. In all the cases described so far, comprehensive immunological study has not identified any preexisting immunological disorder except for the anti-GM-CSF autoantibodies, linking these antibodies to relatively narrow immunodeficiencies. These infections have all had in common neurological involvement. It is noteworthy that in anti-GM-CSF-associated cryptococcosis, *C. gattii* is vastly overrepresented compared to its more common relative, *C. neoformans* [13], suggesting a narrow specificity for GM-CSF in neurological immunity towards some pathogens. The mechanisms underlying these specificities are not yet elucidated.

In most cases of anti-GM-CSF autoantibody-mediated infection, pulmonary function is normal at the time of infection diagnosis, excluding significant concomitant PAP at that time. In some cases, however, PAP has occurred years after the first infectious manifestation [12]. Further, when anti-GM-CSF autoantibodies are initially recognized in the setting of autoimmune PAP, accompanying infections are mostly restricted to the respiratory system [14]. Therefore, the phenotypes induced by anti-GM-CSF autoantibodies are variable and not necessarily overlapping. This observation prompted us to examine why nominally the same anti-GM-CSF autoantibody may cause distinct phenotypes in different hosts. In a first step to address this question, we focused on the functional characteristics of the antibodies. We gathered plasma samples from patients with autoimmune PAP, cryptococcal meningitis and disseminated nocardiosis and compared relative amounts of anti-GM-CSF autoantibodies and neutralizing effect on GM-CSF signaling (assessed by STAT5 phosphorylation) in circulating monocytes.

## Materials and methods

### Patients

Blood samples from 41 patients with proven anti-GM-CSF autoantibodies were collected between March 2010 and April 2019 at the Laboratory of Clinical Immunology and Microbiology at the National Institutes of Health (NIH). Twenty-three of these patients were seen at the NIH; blood samples of the others had been referred for screening from collaborating sites in the US or abroad. Normal PBMCs were obtained though the NIH Blood Bank. Written informed consent was provided by all participants or their guardians under appropriate IRB-approved protocols (07-I-0033, 93-I-0119, 93-I-0106, 95-I-0066).

### Anti-GM-CSF autoantibody determination and quantification.

Plasma samples were screened for anti-GM-CSF autoantibodies using a particle-based approach as previously described [15]. Briefly, fluorescent magnetic beads (Bio-Rad; MC1-0029-01) were covalently coupled to 2.5 μg recombinant human GM-CSF (R&D Systems; catalog 215-GM-050/CF). Beads were incubated with plasma samples for 30 min, washed, then incubated with PE-labeled goat anti-human IgG (ThermoScientific) for an additional 30 min before being run on the Bio-Plex X200 instrument (Bio-Rad). A standard curve was obtained by running on each plate a monoclonal mouse anti-human GM-CSF antibody (R&D Systems) with serial dilutions ranging from 50 to 0.7125 ng/ml (R^2^ of interpolation slopes > 0.98), secondarily revealed by a PE-labeled goat anti-mouse IgG (ThermoScientific). The relative amount of antibodies in patient samples was determined by the mean fluorescence intensity (MFI) of samples compared to those of the standard curve. Because of the use of a mouse monoclonal antibody as standard instead of purified human polyclonal antibodies, we chose to express the concentration results in an arbitrary Mouse Monoclonal Antibody Unit (MMAU). Each patient sample was tested on three distinct occasions with at least three different dilutions between 1/5,000 and 1/500,000 and results from the most reproducible dilution fitting the most points of the standard curve were averaged.

### Plasma isolation, cell culture, and stimulation

Plasma from each subject was separated from whole blood and stored at − 80 °C until testing. Effort was made to choose samples from dates distant from interventions that might influence antibody levels (e.g., rituximab treatments) and before lung transplantation, where relevant.

PBMCs from healthy donors were obtained by density-gradient centrifugation, frozen in media at − 80 °C. Thawed cells were cultured at 10^6^ cells/ml in complete RPMI 1640 (Life Technologies BRL; with 2 mM glutamine, 20 mM HEPES, 0.01 mg/ml penicillin/streptomycin) and 10% fetal calf serum (FCS). For each set of experiments, PBMCs from the same healthy donor were used in order to maintain a constant cellular background.

### Neutralizing activity of anticytokine antibodies.

Neutralizing activity of plasma against GM-CSF was performed by assessing STAT5 phosphorylation in cells stimulated with GM-CSF in the presence of plasma from patients or controls. Briefly, PBMCs (1 × 10^6^ cells/ml) were cultured in complete RPMI 1640 with 10% FCS with plasma from normal subjects or patients and left unstimulated or stimulated with recombinant human GM-CSF (Peprotech) for 30 min at 37 °C. Monocytes were identified by CD14 (BD Pharmingen) surface staining before being fixed and permeabilized for intracellular staining with p-STAT5 (Y694) Ab (BD Bioscience), as described previously [3].

In order to approximate the IC_50_ (concentration of antibodies responsible for 50% of inhibition of GM-CSF-induced STAT5 phosphorylation), the patient plasmas were used at different dilutions (diluted in AB serum from 0.01 to 30%) with a constant concentration of GM-CSF for stimulation (10 ng/ml).

In order to assess the EC_50_ (concentration of GM-CSF responsible for 50% of STAT5 phosphorylation in the presence of antibodies), the volume of plasma was chosen to get a constant final concentration of antibodies in each tube at 10 MMAU (completed with AB serum); stimulation with GM-CSF varied from 10^–1^ to 10^4^ ng/ml.

Data were collected using a BD LSR Fortessa (BD Biosciences), analyzed using FlowJo software (TreeStar), and graphed with Prism8 (GraphPad).

### Statistics

Differences between groups were evaluated using unpaired Student’s *t* test. Correlation analyses were done calculating nonparametric Spearman’s correlation coefficient. The threshold for statistical significance was set to p < 0.05.

## Results

### Patients

We identified 15 patients with circulating anti-GM-CSF autoantibodies and PAP only, 15 patients with cryptococcal meningitis (6/15 confirmed C. *gattii* species) and 5 patients with severe nocardiosis. Six patients had overlapping phenotypes at the time of sampling: 3 with both PAP and cryptococcal meningitis, 2 with PAP and disseminated nocardiosis and 1 patient had sequential nocardiosis then cryptococcosis. In patients with PAP and infections, the infection had preceded the PAP for several years except for one patient who presented PAP and Cryptococcus meningitis concomitantly. The clinical cases of some of these patients have already been reported [11, 12, 16, 17]. Patient characteristics are summarized in Table 1.

**Table 1:** Characteristics of patients with anti-GM-CSF autoantibodies

	N	Sex F/M	Seen in NIH	Age at sampling (years) Median [range]	Anti-GM-CSF aB level (MMAU) Mean ±SD	
PAP alone	15	9/6	11/15	48.1 [30.3–66.4]	495±464	6 Rituximab 2 Lung transplant
*Cryptococcus *infection alone	15	10/5	5/15	35.4 [20.4–75]	197±159	4 *C neoformans* 6 *C. gattii* 5 undetermined
*Nocardia* infection alone	5	2/3	3/5	52.3 [43.7–62.4]	430±493	1 lung only 4 lung +brain
PAP+ *cryptococcus* infection	3	1/2	3	21/44/51		2 *C. neoformans * 1 *C. gattii*
PAP+ *Nocardia *infection	2	0/2	1	44/NA		
*Crypto* + *Nocardia* infection	1	1/0	0	60		

*PAP* Pulmonary Alveolar Proteinosis, *NA* Not available

### Anti-GM-CSF autoantibody levels are higher in PAP than in cryptococcal meningitis

**Fig. 1:**
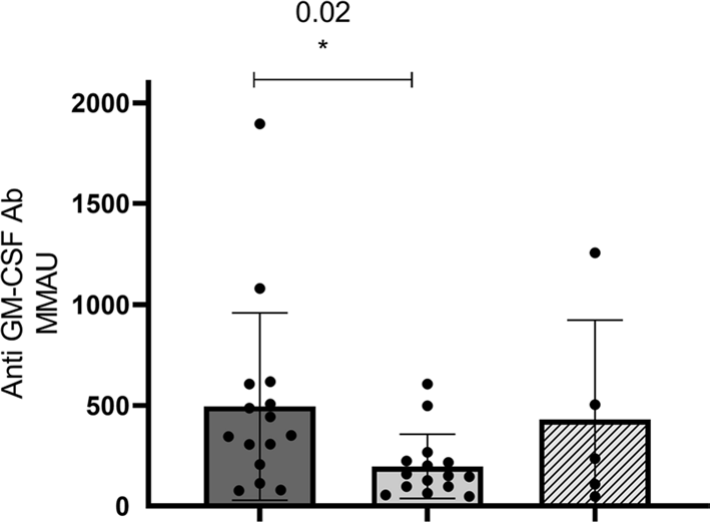
Relative amounts of anti-GM-CSF antibodies. The relative amounts of antibodies were determined using a particle-based approach with GM-CSF coupled fluorescent magnetic beads (15), using a mouse monoclonal anti-GM-CSF as standard. Results for groups of patients with distinct phenotypes are expressed as mean ± SD in an arbitrary Mouse Monoclonal Antibody Unit (MMAU). Comparisons between groups were done using unpaired Student’s t-test

The relative amounts of autoantibody were determined in patient plasmas using a particle-based approach with GM-CSF-coupled magnetic beads and compared between groups (Table 1 and Fig. 1). We restricted the comparison to patients with non-overlapping phenotypes and chose samples from the dates closest to disease diagnosis. The results were expressed in an arbitrary Mouse Monoclonal Antibody Unit (MMAU), according to the experimental protocol. The only significant difference was observed between PAP patients and those with cryptococcal meningitis (mean ± SD: 495 ± 464 MMAU *vs* 197 ± 159 MMAU, *p* = 0.02). No statistical difference was observed between PAP patients and subjects with *Nocardia* infection alone (495 ± 464 MMAU *vs* 430 ± 493 MMAU, p = 0.8) or between subjects with cryptococcosis and *Nocardia* infections (197 ± 159 MMAU *vs* 430 ± 493 MMAU, p = 0.11).

### Global effect of patient plasma on GM-CSF-induced STAT5 phosphorylation: IC_50_

**Fig. 2:**
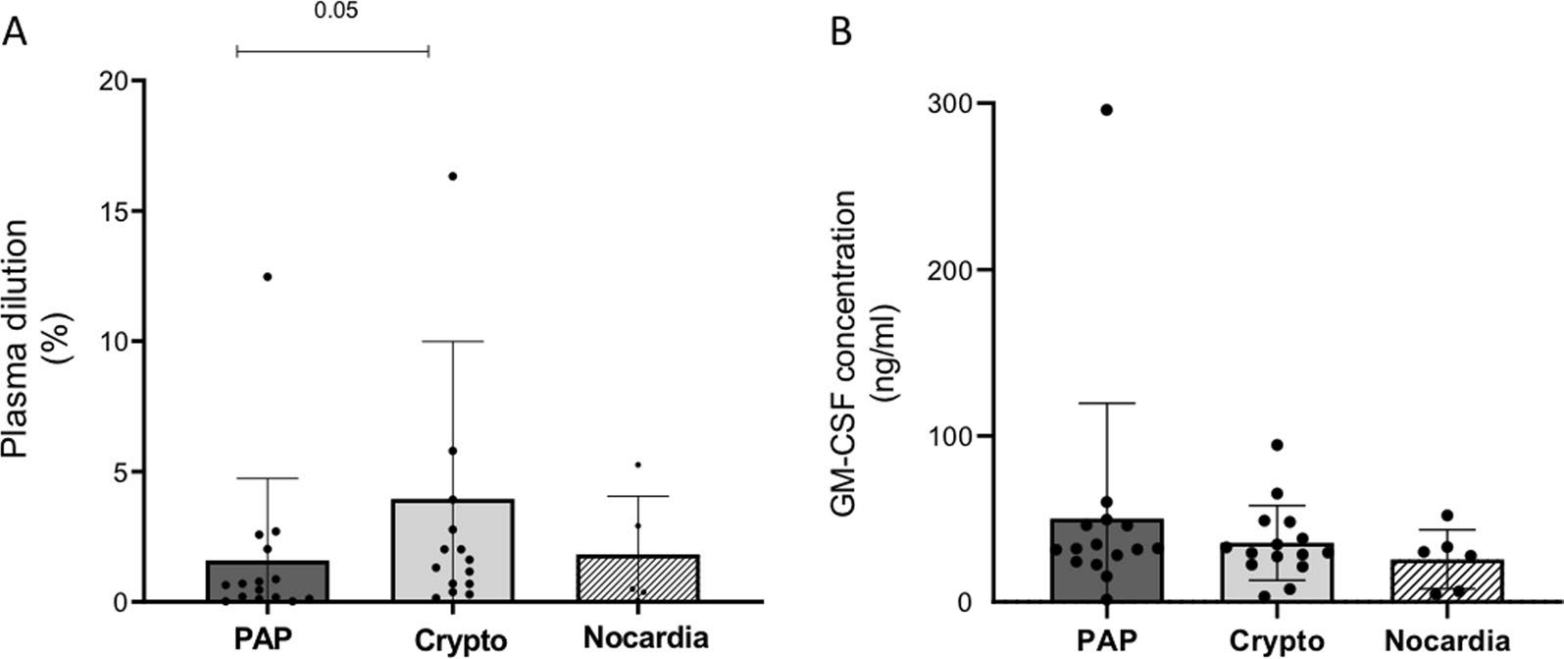
Neutralizing effect of patient plasma on GM-CSF-induced STAT5 phosphorylation: IC_50_ **A** and EC_50_ (**B**). PBMCs from the same healthy donor were cultured with plasma from patients and stimulated with recombinant human GM-CSF. After 30 min at 37 °C, cells were fixed and permeabilized for intracellular pSTAT5 staining in CD14 positive cells. **A** IC_50_ experiments: The dilutions of patient plasma varied from 0.01 to 30% (diluted in AB serum) and the concentration of GM-CSF stayed constant (10 ng/ml). The dilution at which 50% inhibition of the GM-CSF-induced pSTAT5 occurred was determined for each plasma. **B** EC_50_ experiments: The concentration of antibodies was normalized to 10 MMAU for each sample whereas the concentration of GM-CSF for stimulation varied ween 10^–1^ to 10^4^ ng/ml. The concentration of GM-CSF responsible for 50% of maximal GM-CSF-induced pSTAT5 in presence of 10 MMAU of anti-GM-CSF antibody was assessed for each sample. Results for groups of patients with distinct phenotypes are expressed as mean ± SD and comparisons between groups were done using nonparametric *t*-test

To assess the ability of patient plasma to inhibit GM-CSF signaling due to anti-GM-CSF autoantibodies, we evaluated intracellular phosphoSTAT5 (pSTAT5) in circulating monocytes from healthy donors after a fixed dose of GM-CSF (10 ng/ml) in the presence of varying dilutions of patient plasmas (ranging 0.01% to 30%). The global neutralizing effect of patient plasma (dilution of plasma sufficient for 50% inhibition of GM-CSF-induced pSTAT5) tended to be higher in patients with PAP compared to patients with cryptococcal infections: mean ± SD dilution of 1.6 ± 3.1% vs 3.9 ± 6%, *p* = 0.05. The plasma dilution sufficient for 50% inhibition of GM-CSF-induced pSTAT5 was negatively correlated with the antibody relative amounts (r = -0.88), meaning that the higher the dilution the more neutralizing the plasma. Anchoring the plasma dilution to the antibody level determined in each plasma, we calculated the IC_50_ as the concentration of anti-GM-CSF autoantibody resulting in 50% inhibition of GM-CSF-induced pSTAT5. This latter was not significantly different between the groups of patients: mean ± SD of 2.46 ± 2.3 MMAU for PAP patients, 3.7 ± 3.3 MMAU for cryptococcosis patients and 1.94 ± 1.1 MMAU for patients with *Nocardia* infections (Fig. 2A).

### Effect of anti-GM-CSF antibodies on GM-CSF induced STAT5 phosphorylation: EC_50_

As the global neutralizing effect was dependent on antibody level in plasma, we looked at another way to evaluate the inhibitory effect of the antibody itself. To do this, we used a constant concentration of antibody (brought down to 10 MMAU) and varied the GM-CSF amount (10^–1^ to 10^4^ ng/ml) to stimulate normal circulating monocytes. The effect of GM-CSF on STAT5 phosphorylation in monocytes in the presence of a constant antibody concentration led to determination of an EC_50_ for each sample. The mean EC_50_ was not significantly different between groups of patients with different phenotypes: 50.08 ± 69.5 ng/ml in PAP patients, 35.5 ± 22.4 ng/ml in patients with cryptococcal infections and 25.6 ± 18 ng/ml in patients with *Nocardia* infections (Fig. 2B).

### Longitudinal samples

In order to determine changes over time in the quantity or quality of the autoantibodies, we examined the longitudinal samples available for 2 adult patients.

**Fig. 3:**
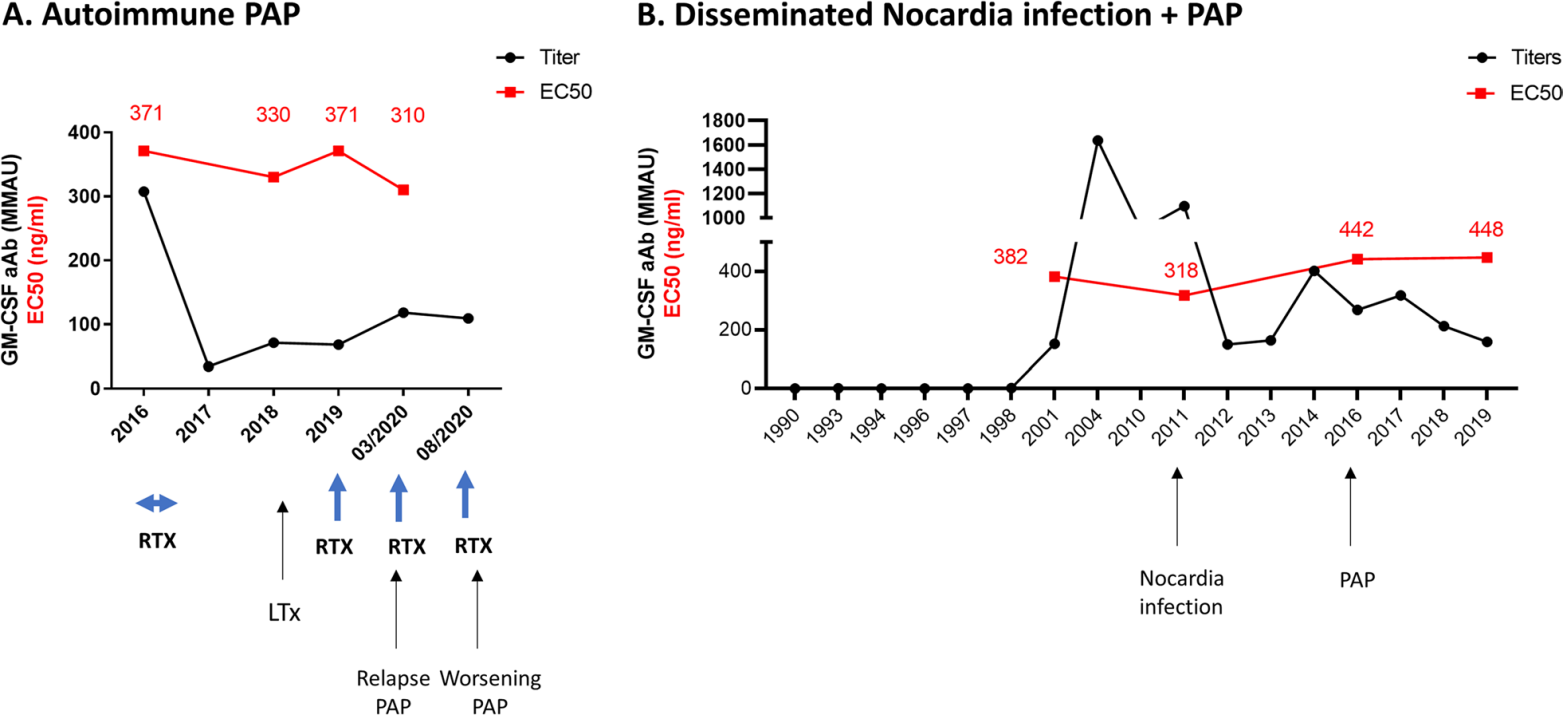
Evolution of anti-GM-CSF autoantibody levels and neutralizing activity (EC_50_). **A** Patient with autoimmune PAP receiving rituximab pulses and lung transplantation. **B** Patient treated for a disseminated *Nocardia* infection who developed PAP 5 years later. The neutralizing activity of the antibodies was assessed by the EC_50_: the concentration of GM-CSF responsible for 50% of the maximal GM-CSF-induced pSTAT5 in the presence of plasma containing 10 MMAU of anti GM-CSF antibody. *RTX* rituximab, *LTx* lung transplantation, *PAP* pulmonary alveolar proteinosis

The first one was a man referred in 2016, 8 years after the diagnosis of an autoimmune pulmonary alveolar proteinosis which progressed despite 14 whole lung lavages (Fig. 3A). Six months of rituximab and inhaled and subcutaneous GM-CSF reduced his anti-GM-CSF autoantibodies dramatically. However, his respiratory status deteriorated and he underwent double lung transplantation in July 2018. Circulating anti-GM-CSF levels rose post-transplant despite immunosuppressive therapy (corticosteroids, mycophenolate mofetil and tacrolimus) and rituximab pulses. Recurrent PAP in the transplanted lungs was identified in March 2020 on CT scan and his respiratory status worsened 6 months later. Despite multiple therapies, and corresponding decreased levels post rituximab therapy, his neutralizing anti-GM-CSF autoantibodies remained qualitatively constant over time (EC_50_ of patient plasma on GM-CSF induced pSTAT5 phosphorylation in normal monocytes) (Fig. 3A).

The second patient was a man diagnosed with disseminated *Nocardia* infection in 2011 involving the lungs and the brain (previously reported in ref 11) (Fig. 3B). Banked plasma going back until 1990 were available and high level anti-GM-CSF autoantibodies were identified 10 years before this first infectious manifestation. *Nocardia* therapy was successful but 5 years after initial presentation, progressive respiratory impairment led to the diagnosis of PAP in 2016. Inhaled GM-CSF therapy improved his respiratory function. His EC_50_ over time remained high despite variation in anti-GM-CSF antibodies levels and evolving clinical manifestations.

## Discussion

Anti-GM-CSF antibodies have been described in various and not necessarily overlapping clinical situations. We queried whether the characteristics of the anti-GM-CSF antibodies (in terms of levels and neutralizing activity) varied between patients with autoimmune PAP and those with autoantibody-related severe infections. We found that (i) patients with autoimmune PAP tended to have higher levels of anti GM-CSF autoantibodies, (ii) levels and global neutralizing activity of plasma correlated, and (iii) there was no significant difference between groups in terms of neutralizing activity of anti-GM-CSF autoantibodies.

The disease induced by anti-cytokine antibodies is the result of a complex combination of several factors including the characteristics of the antibody (e.g., epitope, level, Ig subclasses, avidity), level of patient exposure to certain irritants and pathogens and potentially individual genetic susceptibility. Characteristics of the antibody have been shown to contribute to disease activity and phenotype in other autoantibody-mediated diseases [18, 19]. Anti-GM-CSF antibodies have been extensively studied in the context of autoimmune PAP. A genetic predisposition to the appearance of anti-GM-CSF antibodies has long been suspected; genetic studies have provided conflicting results depending on ethnicity or techniques used so far. [20, 21]. Serum anti-GM-CSF autoantibody levels, unlike levels in bronchoalveolar fluid, do not correlate with disease severity in patients with autoimmune PAP [22, 23]. It seems that a “critical threshold” (5 μg ml^–1^) exists above which anti-GM-CSF antibodies are likely to disrupt GM-CSF-stimulated functions in alveolar macrophages and blood leukocytes [24, 25] and are strongly associated with an increased risk of autoimmune PAP [26]. Monoclonal anti-GM-CSF antibodies from patients with autoimmune PAP have been isolated from cloned memory B cells. The authors identified several clones, mainly of the IgG1 subtype, targeting multiple non-overlapping epitopes and utilizing multiple immunoglobulin variable region V genes [27, 28]. Further, the molecular and crystal structures of anti-GM-CSF antibodies have been solved [29–31]. The targeted epitopes can be linked to differences in affinity [27]. The antibody binding to GM-CSF is further refined by somatic mutations [28]. It appears that it takes a combination of three or more non cross-competing antibodies to effectively neutralize GM-CSF activity in vitro and in vivo, while a single anti-GM-CSF neutralizing monoclonal antibody may not be harmful [28]. Piccoli et al., showed that injection of a cocktail of different antibodies could lead in vivo to the formation of high molecular weight complexes sequestering GM-CSF and promoting the rapid degradation of GM-CSF-containing immune complexes in a Fc-dependent manner [28]. Taken together, these observations show that (i) anti-GM-CSF autoantibodies in autoimmune PAP are composed of polyclonal IgG, (ii) suggest that their formation is driven by GM-CSF itself, (iii) that the memory B cell maturation process is helped by T cells, implying that somatic mutations determine antibody affinity, and (iv) are reassuring that treatments based on single anti-GM-CSF monoclonal antibodies that are currently in development for inflammatory diseases may not necessarily cause PAP or infectious disease by themselves [32, 33].

Our study is the first to address and compare anti-GM-CSF antibody characteristics in diseases other than autoimmune PAP. Previous reports have noticed a predominance of IgG1 subclass antibodies in patients with *Cryptococcus* or *Nocardia* infections, as is true for autoimmune PAP [11, 12]. GM-CSF secretion is induced during infectious or inflammatory processes; infectious disease preceding autoimmune PAP diagnosis may have suggested a pathogen-induced autoimmunity mechanism in some cases [12, 34]. However, the demonstration of high-level anti-GM-CSF autoantibodies 10 years before CNS nocardiosis and 15 years before autoimmune PAP in one patient renders unlikely this hypothesis. What, if anything, distinguishes anti-GM-CSF autoantibody patients with *Cryptococcus* or *Nocardia* infection from each other and from patients with PAP alone remains obscure. Although our study was not designed to determine absolute levels of antibodies, it allowed us to compare the relative amounts and activities of autoantibodies in the plasmas of these three groups of patients. No dramatic differences were found between groups, except for a slightly higher level in PAP patients. The in vitro neutralizing effects of anti-GM-CSF autoantibodies were not statistically different by phenotype. Plasma from PAP patients tended to be more effective at blocking GM-CSF signaling (effective at 1.6% dilution) than plasma from patients with cryptococcosis (3.9%, over 2 times more). The use of whole plasmas cannot exclude other possible non-antibody inhibitory effects. However, anchoring the plasma dilution to the antibody level, we did not find any difference in IC_50_ determined as the concentration of anti-GM-CSF autoantibody responsible for 50% inhibition of GM-induced pSTAT5. We also did not find it regarding the EC_50_ for pSTAT5 experiments. For each set of experiments, PBMCs from the same healthy donor were used in order keep a constant genetic background underlying the cellular response.

These in vitro effects may not perfectly reflect the neutralizing activities of the antibodies in vivo [28]. Further studies on the kinetics and structures of the antibodies in these three groups of patients are warranted. Alternative hypotheses could include tissue-restricted specificities. Anti-GM-CSF antibodies may act differently on pulmonary macrophages than circulating monocytes. Therefore, the lack of difference between the antibodies from these three groups, studied solely on circulating monocytes, may not accurately reflect what happens in alveolar spaces. Indeed, human circulating monocytes and monocyte-derived macrophages differ in various cellular functions and do not accurately recapitulate the biology of human lung macrophages [35, 36]. Macrophages from the central nervous system are also distinct from circulating monocytes and monocyte-derived macrophages. Finally, it may be that the antibodies are indeed all essentially the same and some other aspect of underlying host experience or genetics is the determinant of the clinical manifestation. Cigarette smoking and dust exposure have been suggested as causal or aggravating factors for autoimmune PAP, by impairing lung capillaries permeability for example and favoring the leaking out of antibodies into the alveolar space [37, 38]. Comprehensive genome sequencing in all affecteds is underway to address possible genetic susceptibilities.

Almost every case of anti-GM-CSF–related disseminated infection in our cohort involved the CNS. The search for anti-GM-CSF antibodies is now recognized as part of the workup in “non-immunocompromised” patients with brain infection with *Cryptococcus* or *Nocardia* [39, 40].

Recent studies suggest that GM-CSF, elicited by IL-23-driven T cells, favors CNS invasion by phagocytes and secondarily myeloid-mediated tissue immunopathology [41–43]. Anti-GM-CSF antibodies are detectable in the cerebrospinal fluid of patients with cryptococcal meningitis, albeit at lower levels than in the circulation [11]. To what extent these autoantibodies disrupt local immunity against fungal or bacterial pathogens and whether they predispose to CNS infection specifically needs further exploration.

Longitudinal samples may be particularly informative. In our patients who developed different manifestations of anti-GM-CSF autoantibodies, the infections usually preceded PAP. In only one patient was PAP diagnosed concomitant with cryptococcal meningitis. Globally, PAP seems to occur later in clinical evolution. As it does not appear to be a matter of circulating antibody levels or global neutralizing effect, this temporality could be due to cumulative effects over time or changes in exposure.

Our study was retrospective, gathering samples from different centers; some clinical data may have been overlooked. The precise date of onset of the initial manifestation may have been missed. Additionally, the delay between diagnosis and date of sampling was variable between patients, with some sampling made at intervals from onset and after several lines of treatment. These realities make the value of our rare longitudinal samples greater. Regardless of these shortcomings, which might serve to artifactually amplify differences, we found very few.

## Conclusion

This study is the first to gather and compare cohorts of patients with the various anti-GM-CSF antibody-related clinical manifestations of PAP, cryptococcal meningitis, and disseminated *Nocardia* infection. We found no explanatory differences between the groups in terms of relative amounts of circulating antibody or in vitro neutralizing effect on GM-CSF-induced STAT5 phosphorylation in monocytes. Genetic analysis, affinity assays on various cellular targets and longitudinal samples will be needed to help delineate the biology of these anti-GM-CSF autoantibodies.

## Data Availability

The comprehensive data are available upon request to the corresponding author.

## Abbreviations

aAb: Auto antibodies
GM-CSF: Granulocyte–macrophage-colony-stimulating-factor
MFI: Mean fluorescence intensity
PAP: Pulmonary alveolar proteinosis
pSTAT5: Phospho-STAT5
CNS: Central Nervous System
